# Phthalate Excretion Pattern and Testicular Function: A Study of 881 Healthy Danish Men

**DOI:** 10.1289/ehp.1205113

**Published:** 2012-07-24

**Authors:** Ulla Nordström Joensen, Hanne Frederiksen, Martin Blomberg Jensen, Mette Petri Lauritsen, Inge Ahlmann Olesen, Tina Harmer Lassen, Anna-Maria Andersson, Niels Jørgensen

**Affiliations:** 1 Department of Growth and Reproduction, and; 2 Fertility Department, Copenhagen University Hospital, Copenhagen, Denmark

**Keywords:** DEHP, DiNP, male reproduction, phthalates, semen quality, testosterone, %MEHP, %MiNP

## Abstract

Background: In animals, some phthalates impair male reproductive development and function. Epidemiological studies have reported inconsistent evidence of associations between phthalates and markers of human testicular function.

Objectives: We aimed to provide estimates of the effects of phthalate exposure on reproductive hormone levels and semen quality in healthy men.

Methods: A total of 881 men gave urine, serum, and semen samples. Serum levels of testosterone, estradiol (E_2_), sex hormone-binding globulin (SHBG), luteinizing hormone (LH), follicle-stimulating hormone (FSH), and inhibin-B; semen quality; and urinary concentrations of 14 phthalate metabolites, including metabolites of di(2-ethylhexyl) phthalate (DEHP) and diisononyl phthalate (DiNP), were assessed. The proportions of DEHP and DiNP excreted as their respective primary metabolites [mono(2-ethylhexyl) phthalate (MEHP) and mono-isononyl phthalate (MiNP)] were calculated and expressed as percentages (%MEHP and %MiNP, respectively).

Results: The free androgen index was 15% lower [95% confidence interval (CI): –23, –8%] for men in the highest %MiNP quartile compared to the lowest quartile (*p* < 0.001) after adjusting for confounders, and 9% lower (95% CI: –16, –1%) in the highest %MEHP quartile (*p* = 0.02). %MEHP and %MiNP were negatively associated with the ratio of testosterone/LH and testosterone/FSH. %MEHP was negatively associated with total testosterone, free testosterone, and ratio of testosterone/E_2_. %MiNP was positively associated with SHBG. There was little evidence of associations between urinary phthalate metabolites or sums of phthalates with reproductive hormones or semen quality

Conclusion: Our data suggest that both testosterone production and pituitary–hypothalamic feedback may be compromised in individuals excreting a high proportion of primary metabolites of long-chained phthalates relative to the proportion of secondary metabolites.

There is concern that environmental exposures may impair male reproductive function, and epidemiological studies support an inverse association between phthalate exposure and markers of testicular function. This has been shown in occupationally exposed men (Pan et al. 2006), fertile men (Mendiola et al. 2011), men from an infertility clinic (Duty et al. 2003, 2005; Hauser et al. 2006; Meeker et al. 2009), and healthy young men (Jönsson et al. 2005) although another study of men from an infertility clinic found no significant associations (Herr et al. 2009). Fertile, infertile, and occupationally exposed men are selected groups; to date there has been only one smaller study of such associations in an unselected population of 234 men (Jönsson et al. 2005).

Humans are exposed to diester phthalates, which are metabolized into monoesters and mainly excreted in urine. Di(2-ethylhexyl) phthalate (DEHP) is metabolized to the primary metabolite mono(2-ethylhexyl) phthalate (MEHP) and subsequently to secondary metabolites. Both primary and secondary metabolites can be measured in urine, and a sum of excreted DEHP metabolites can be calculated. The percentage of DEHP excreted as the primary metabolite, MEHP, is termed %MEHP (Hauser 2008). %MEHP as a marker of DEHP metabolism has previously been associated with increased sperm DNA damage (Hauser et al. 2007) and altered reproductive hormones (Meeker et al. 2009). Similarly to DEHP, diisononyl phthalate (DiNP) is excreted as a primary metabolite, mono-isononyl phthalate (MiNP), along with secondary metabolites. %MiNP as a marker of DiNP metabolism has, to our knowledge, not previously been studied in relation to any outcome.

Some authors have noted evidence of decreasing testosterone levels that coincides with evidence of a simultaneous decline in semen quality during the 20th century (Andersson et al. 2007; Carlsen et al. 1992; Jørgensen et al. 2011). These changes appear to have occurred over a relatively short period, consistent with effects due to changes in lifestyle or environmental factors, rather than genetic influences. Animal studies have established that some phthalates, including di-*n*-butyl phthalate (DnBP), DEHP, and DiNP, act as endocrine disruptors that affect both Leydig cell and germ cell development, resulting in developmental abnormalities of the reproductive tract and inhibition of testicular testosterone production in pre- or perinatally exposed animals (Boberg et al. 2011; Gray et al. 2000). Humans may also be affected by phthalates early in life (Main et al. 2006; Suzuki et al. 2012; Swan et al. 2005), consistent with the testicular dysgenesis syndrome (TDS) hypothesis (Skakkebæk et al. 2001) that posits a common fetal origin of some cases of male reproductive disorders.

Potential mechanisms for the effects of phthalates on human adult testicular function have not been clearly established. However, a recent study showed that DEHP and MEHP can inhibit testosterone synthesis in cultured adult human testicular tissue, with MEHP concentrations estimated to be within the range observed in epidemiological studies (Desdoits-Lethimonier et al. 2012). Phthalates are not persistent or bioaccumulative chemicals, but humans are continuously exposed throughout life to several phthalates through the diet and products such as cosmetics, perfume, paints, and plastics, especially polyvinyl chloride (PVC) (Wittassek et al. 2011).

In the present study, we describe associations between urinary phthalate excretion and reproductive hormones and semen quality, and estimate the effects of exposure to different phthalates in a large unselected group of healthy young men from the general population.

## Materials and Methods

*Study population.* A total of 900 young Danish men from the general population participated in 2007–2009 in an ongoing semen quality study and each provided a spot urine sample. Out of the 900 men, 19 were excluded: 1 because of a history of testicular cancer; 1 because of testicular cancer discovered at examination; 4 because of self-reported abuse of anabolic steroids; and 2 because of grossly abnormal hormone levels but normal virilization and testis size at physical examinations, strongly indicating anabolic steroid abuse. We further excluded 11 men because of missing blood or semen samples. Thus, the final study population included 881 men (2007: *n* = 287; 2008: *n* = 298; and 2009: *n* = 296).

Basic study details have previously been described (Ravnborg et al. 2011). The men underwent a physical examination, handed in a questionnaire, provided a spot urine sample and a semen sample, and had a blood sample drawn, in most cases all within 1 hr. Ejaculation abstinence period and time of blood sampling were recorded. All semen, urine, and blood samples were collected between 0840 hours and 1230 hours (median 1000 hours).

The research protocol was approved by the Danish National Committee on Biomedical Research Ethics, Copenhagen, Denmark (no. H-KF-289428). Participants gave written informed consent before participation.

*Questionnaire.* Questions included information on lifestyle and medical history. Responses were reviewed together with the participant to clarify missing or unclear information. Participants gave self-reported information on use of medication for ≥ 7 consecutive days within the past 3 months, but were not asked to further specify when the medication was taken. Thus, a participant may have reported taking medication within the previous 3 months, but still have taken no medication at all within the past ≥ 24 hr. Ethnicity was classified based on the self-reported country of birth of the participant and his parents. Alcohol intake was calculated as the sum of reported alcohol units ingested on each day within the week prior to participation. The men were informed that one beer, one glass of wine, or 40 mL of spirit contained 1 unit of alcohol; one strong beer or one “alcopop,” contained 1.5 units; and one bottle of wine contained 6 units.

*Urinary phthalate metabolite analyses.* Spot urine samples were collected in polyethylene cups, and 15 mL was decanted to a 20-mL glass scintillation vial with the tops packed with aluminum foil and stored at –20°C until analysis. Analysts were blind to any information regarding the subjects. Samples were analyzed for content of 14 phthalate metabolites: monoethyl phthalate (MEP), mono-*n*-butyl phthalate (MnBP), mono-isobutyl phthalate (MiBP), monobenzyl phthalate (MBzP), MEHP, mono(2-ethyl-5-hydroxyhexyl) phthalate (MEHHP), mono(2-ethyl-5-oxohexyl) phthalate (MEOHP), mono(2-ethyl-5-carboxypentyl) phthalate (MECPP), mono-*n*-octyl phthalate (MOP), mono(3-carboxypropyl) phthalate (MCPP), MiNP, mono(hydroxy-isononyl) phthalate (MHiNP), mono(oxo-isononyl) phthalate (MOiNP) and mono(carboxy-isooctyl) phthalate (MCiOP) by liquid chromatography tandem mass spectrometry (LC-MS/MS) with preceding enzymatic deconjugation followed by solid phase extraction. The method for sample preparation, standard solutions, quality controls, instrumental analysis and general method validation has been described previously (Frederiksen et al. 2010).

In the present study, urine samples were analyzed in 25 batches over 11 weeks. Each batch included standards for calibration curves, around 35 unknown samples plus 2 blanks, 2 urine pool controls, and 2 urine pool controls spiked with phthalate standards in low levels. The interday assay variation, expressed as the relative SD, was < 20% for all analytes except MCPP (22%) and MiNP (26%). Recovery of spiked control samples was > 90% for all analytes except MnBP (88%) and MCPP (85%). Limits of detection (LODs) were established as previously described (Frederiksen et al. 2010) and were 0.46 ng/mL (MEP), 0.55 ng/mL (MiBP), 0.57 ng/mL (MnBP), 0.25 ng/mL (MBzP), 0.63 ng/mL (MEHP), 0.30 ng/mL (MEHHP), 0.30 ng/mL (MEOHP), 0.17 ng/mL (MECPP), 0.15 ng/mL (MOP), 0.14 ng/mL (MCPP), 0.15 ng/mL (MiNP), 0.12 ng/mL (MHiNP), 0.11 ng/mL (MOiNP), and 0.05 ng/mL (MCiOP). Results below LOD were assigned a value of LOD ÷ square root of 2.

We calculated the sum of dibutyl phthalate (DBP) isomers [ΣDBP_(i+_*_n_*_)_] and sums of DEHP and DiNP metabolites (ΣDEHPm and ΣDiNPm) as described previously (Frederiksen et al. 2010). For individuals with detectable levels of MEHP, we also calculated the percentage of total ΣDEHPm excreted as MEHP, referred to as %MEHP (Hauser 2008) [%MEHP = MEHP (nanomoles per milliliter)/ΣDEHPm (nanomoles per milliliter)], and similarly calculated %MiNP as the percentage of total ΣDiNPm excreted as MiNP. Molar sums of the metabolites of low-molecular-weight phthalates (MEP, MiBP, and MnBP) and metabolites of high-molecular-weight phthalates (MBzP, MCPP, and DEHP and DiNP metabolites), and the molar sum of all phthalate metabolites, were calculated as described by Wolff et al. (2010).

We did not adjust for urine dilution by specific gravity because it has been shown previously that this does not substantially influence phthalate metabolite concentrations (Duty et al. 2005). Similarly, creatinine-adjusted metabolite concentrations are correlated closely with unadjusted concentrations (Jönsson et al. 2005). In addition, %MEHP and %MiNP represent relationships between urinary concentrations of metabolites that are independent of urinary dilution.

*Reproductive hormone analyses.* Blood samples were drawn from the cubital vein. Serum was stored at –20°C until analysis, and subsequently analyzed in batches: June–August 2008 (*n* = 293), August 2009 (*n* = 33), and June 2010 (*n* = 555). Analysts were blind to any information regarding the subjects. Levels of follicle-stimulating hormone (FSH), luteinizing hormone (LH), and sex hormone–binding globulin (SHBG) were measured by time-resolved flouroimmunoassay (Delfia, Wallac, Turku, Finland). Testosterone (total testosterone) and estradiol (E_2_) were determined by radioimmunoassay (Coat-a-Count; DPC, Los Angeles, CA, USA, and Pantex, Santa Monica, CA, USA). Inhibin-B was determined by a double antibody enzyme-immunometric assay. The intra- and interassay coefficients of variation (CV) for measurement of FSH, LH, and SHBG were < 6%, and CVs for total testosterone were < 10%. Intra- and interassay CVs were 8% and 13% for estradiol and 15% and 18% for inhibin-B, respectively. Samples were analyzed for inhibin-B using kit material from Oxford Bio-Innovation Ltd. (Bicester, UK) in 2008, or kit material from Beckman Coulter Inc. (Webster, TX, USA) in 2010. Between 2008 and 2010, slight adjustments were made to the inhibin-B analysis, resulting in a lower LOD (7 pg/mL) in 2010 than in 2008 (20 pg/mL).

We calculated the free androgen index (FAI) as [total testosterone × 100/SHBG], and free testosterone from total testosterone and SHBG using a fixed albumin level of 43.8 g/L as described by Vermeulen et al. (1999). Hormone ratios were calculated by simple division.

*Semen analysis.* Semen volume was assessed by weight, and sperm concentration was determined using a Bürker-Türk hemocytometer (Paul Marienfeld GmbH & Co. KG, Lauda-Königshofen, Germany). Total sperm count (semen volume × sperm concentration) and percentage of progressively motile spermatozoa [World Health Organization (WHO) class A + B] were calculated (WHO 2010). Morphology slides were fixed and Papanicolaou-stained and assessed according to strict criteria (Menkveld et al. 1990). Semen analysis was performed in accordance with WHO guidelines as described in detail previously (Jørgensen et al. 2002). Technicians had no access to any information regarding the participants.

*Statistics.* Basic descriptive statistics were derived for population characteristics, serum levels of reproductive hormones, semen parameters, and urinary phthalate levels. Correlations between phthalate metabolite concentrations, as well as possible confounders, were explored using Spearman correlations. We constructed quartile variables for individual phthalate metabolites, summed metabolites, and %MiNP and %MEHP that were entered as fixed ordinate variables coded using integer values (1–4) in multivariate linear regression models. Phthalate metabolites were also modeled as continuous variables. Dependent variables were ln transformed (all hormones, hormone ratios, and semen volume), cubic-root transformed (sperm concentration, total sperm count), squared (progressively motile), or square-root transformed (morphologically normal) to achieve normality of distribution of the residuals. Adjusted means and 95% confidence intervals (CIs) were calculated by back-transformation of estimates from linear regression analysis. *p*-Values for linear trend across quartiles were derived from analyses entering phthalate quartile variables as ordinal categorical coded using integer values (1–4).

In models of associations between hormone levels and phthalate metabolite concentrations, the following covariates were statistically significant predictors (*p* < 0.05) of one or more outcomes: body mass index [BMI; weight (in kilograms) ÷ height (in meters squared)] was negatively associated with total testosterone and SHBG and positively associated with E_2_ nd LH. Smoking (cigarettes per day) was positively associated with total testosterone, E_2_, FAI, and free testosterone. Alcohol consumption (units of alcohol consumed in the week prior to participation) was positively associated with total testosterone and E_2_, FAI, and free testosterone and negatively associated with SHGB. Time of blood sampling was positively associated with LH, but not with total testosterone. Age was positively associated with total testosterone, inhibin-B, and free testosterone. All these potential confounders were included as continuous variables in the models. In addition, models of associations with inhibin-B were adjusted for analysis method as a dichotomous variable. Models of semen volume, concentration, and total count were adjusted for abstinence time (a significant positive predictor) and models of progressively motile sperm were adjusted for time from ejaculation to analysis (a significant negative predictor). Percentage of morphologically normal sperm was left unadjusted because none of the tested covariates were significant predictors of this outcome. Other potential confounders that were considered but not included in the final models because they were not significantly associated with the outcomes were as follows: ethnicity (modeled as seven categories shown in Table 1), BMI squared, *in utero* exposure to tobacco smoke, previous or current diseases [cryptorchidism (treated or untreated), sexually transmitted diseases, varicocele, and inguinal hernia], recent fever, and recent use of medication. Data analysis was performed using PASW Statistics, version 18 (IBM, New York, NY, USA). An alpha level of 0.05 was chosen; *p*-values of < 0.05 were considered statistically significant in all statistical models.

**Table 1:** Characteristics of the study population (*n* = 881).

Characteristic	Mean ± SD	Median (5th, 95th)	*n* (%)
Age (years)	19.5 ± 1.3	19.1 (18.4, 22.0)
Height (cm)	181 ± 6.5	181 (171, 193)
Weight (kg)	75.4 ± 11.6	74.0 (59.5, 95.9)
BMI (kg/m2)	22.9 ± 3.2	22.6 (18.7, 28.7)
Ejaculation abstinence (hours)	75 ± 52	63 (37, 133)
Has (had)
Cryptorchidisma	48 (5.4)
Cryptorchidism treateda,b	20 (2.3)
Sexually transmitted diseasec	73 (8.3)
Varicoceled	103 (11.7)
Inguinal hernia	44 (5.0)
Good or very good general healthe	800 (90.8)
Medication within last 3 monthsf	127 (14.4)
Drank > 21 units of alcohol in last week	224 (25.4)
Daily smoker	362 (41.1)
Mother smoked during pregnancy	232 (26.3)
Fever within recent 3 months	66 (7.5)
Ethnicity
Danish	707 (83.8)
Other European	54 (6.4)
Middle Eastern	32 (3.8)
African	20 (2.4)
Asian	16 (1.9)
Latin American	11 (1.3)
Greenlander	4 (0.4)
Not reported	37
5th and 95th are percentiles. aNot born with both testicles in scrotum (includes spontaneous descent, treated cases, or still cryptorchid). bHormonal or surgical treatment, or combination. cIncluded chlamydia, condylomas, genital herpes, and/or gonorrhea. dDiagnosed previously or on day of participation. eQuestion was “How would you describe your own health? Very good, good, fair or poor.” fTaken medication for skin conditions (n = 33), systemic antibiotics (n = 29), analgesics (n = 29), asthma/allergy medication (n = 27), other medication (n = 16).						

## Results

*Population characteristics.* Participants were healthy young men, around 19 years of age, mostly of Danish origin (participant and both parents born in Denmark). Most men had BMI within the normal range of 18.5–25 kg/m^2^ (median 22.6 kg/m^2^). Of the participants, 59% were nonsmokers and 25% had a high (> 21 units) weekly intake of alcohol. Basic characteristics are shown in Table 1. Reproductive hormone levels and semen quality parameters are shown in Table 2, and were consistent with expected levels in healthy young Danish men.

**Table 2:** Reproductive hormone levels and semen quality parameters of the study population (*n* = 881).

Parameter	Mean ± SD	Median (5th, 95th)	Adjusted mean (95% CI)
Hormone
Total testosterone (nmol/L)	19 ± 6	19 (11, 31)	17 (16, 18)a
SHBG (nmol/L)	29 ± 12	28 (13, 50)	25 (23, 27)a
Estradiol (pmol/L)	80 ± 25	77 (44, 127)	69 (64, 74)a
LH (IU/L)	3.3 ± 1.5	3.1 (1.4, 6.3)	2.6 (2.4, 2.9)a
Inhibin-B (pg/mL)	183 ± 65	175 (94, 304)	173 (158, 188)a
FSH (IU/L)	2.7 ± 1.6	2.3 (1.9, 5.6)	2.2 (1.9, 2.5)a
FAI	75 ± 35	67 (39, 133)	68 (62, 74)a
Free testosterone (ng/dL)	13 ± 4.2	12 (7.6, 20)	11 (11, 12)a
Semen quality
Semen volume (mL)	3.5 ± 1.6	3.2 (1.3, 6.3)	3.6 (3.4, 3.8)b
Sperm concentration (million/mL)	61 ± 54	48 (4.1, 169)	63 (57, 69)b
Total sperm count (million)	196 ± 173	145 (13, 530)	217 (195, 240)b
Progressively motile (%)	59 ± 16	61 (27, 80)	61 (60, 62)c
Morphologically normal (%)	7.6 ± 5.0	7.0 (0.5, 16.5)
5th and 95th are percentiles. aPredicted mean hormone levels for men with BMI of 23, no smoking, no alcohol in last week, blood sample time 0800 hours, and inhibin-B assay in 2010 (inhibin-B values only). bPredicted mean levels for men with abstinence time ≥ 96 hr. cPredicted mean levels for men with a time to semen analysis of 40 min.						

*Phthalate levels.* All phthalate metabolites were detectable in > 95% of participants with the exception of MiNP and MOP, which were detected in 79% and 2.5% of the men, respectively (Table 3). Urinary concentrations of MOP were very low (0.1 ng/mL ± 0.05). MCPP is a metabolite of dioctyl phthalate (DOP), but also a minor metabolite of diisooctyl phthalate (DiOP), diisodecyl phthalate (DiDP), DiNP, DEHP, butylbenzyl phthalate (BBzP), and DBP, making it difficult to relate this metabolite to exposure to any specific phthalate. Therefore, data on MOP and MCPP were not analyzed further. All phthalate metabolites and sums of phthalate isomers and metabolites (ΣDBP_(i+n)_, ΣDEHPm, and ΣDiNPm) were positively correlated (all *p* < 0.001) (data not shown).

**Table 3:** Urinary concentrations (ng/mL) of phthalate metabolites (*n* = 881).

Metabolite	Percent > LOD	Mean ± SD	Median (5th, 95th)	Adjusted mean^a^ (95% CI)
Parent compound
DEP
MEP	100.0	401 ± 1094	78 (11, 1936)	73 (58, 91)
DnBP
MnBP	100.0	36 ± 31	28 (5.6, 91)	21 (18, 23)
DiBP
MiBP	100.0	78 ± 136	58 (12, 173)	55 (49, 62)
BBzP
MBzP	99.8	54 ± 79	34 (5.8, 164)	31 (26, 35)
DEHP
MEHP	94.5	6.7 ± 13	4.0 (0.4, 18)	2.8 (2.4, 3.2)
MEHHP	99.8	39 ± 118	23 (4.3, 79)	18 (15, 20)
MEOHP	99.8	26 ± 79	14 (2.4, 55)	8.3 (7.3, 9.5)
MECPP	100.0	27 ± 81	15 (3.0, 54)	11 (9.7, 13)
DOP
MOP	2.5	0.1 ± 0.05	0.1 (0.1, 0.1)	0.1 (0.1, 0.1)
MCPPb	98.7	7.0 ± 8.5	5.0 (0.7, 20)	3.6 (3.1, 4.2)
DiNP
MiNP	79.6	1.5 ± 4.3	0.6 (0.1, 4.7)	0.5 (0.4, 0.6)
MHiNP	98.7	8.2 ± 17	4.5 (0.5, 23)	3.9 (3.3, 4.6)
MOiNP	98.3	4.1 ± 9.2	2.3 (0.3, 12)	2.0 (1.7, 2.4)
MCiOP	100.0	13 ± 26	7.7 (1.5, 41)	7.2 (6.3, 8.3)
Sums of metabolites (ng/mL)								
∑DBP(i+n)	142 ± 194	110 (22, 315)	95 (85, 107)
∑DEHPm	131 ± 378	75 (15, 260)	54 (48, 62)
∑DiNPm	36 ± 72	21 (3.2, 107)	19 (16, 22)
Percent primary metabolites (%)								
%MiNP	6.2 ± 4.2	5.0 (1.5, 15)	4.5 (4.0, 5.1)
%MEHP	8.7 ± 4.4	8.1 (2.9, 17)	7.5 (6.9, 8.2)
5th and 95th are percentiles. aPredicted mean values for nonsmoking men 19 years of age who provided samples and data on 4 December 2009. bMCPP is a metabolite of several parent phthalates, including DOP, DiOP, DiDP, DiNP, DEHP, BBzP, and DBP.								

Phthalate metabolite levels were weakly positively correlated (all rho ≤ 0.1) with age at participation (all except MEP, MnBP, MEOHP, and MECPP) and number of cigarettes smoked/day (all except MnBP, MiBP, MBzP, MEHP, MEOHP, and MiNP). Levels of phthalate metabolites were generally not correlated with BMI, time of day of urine sample delivery, or alcohol intake (data not shown).

Year of participation was a significant predictor of some diester phthalates in models adjusted for age and smoking, with higher levels of diethyl phthalate (DEP), DnBP, and ΣDEHPm in 2007 than in 2009 (adjusted median ΣDEHPm levels decreased from 80 to 56 ng/mL for participants from 2007 to 2009). However, diisobutyl phthalate (DiBP), BBzP, DiNP, %MEHP, and %MiNP did not differ significantly by year (results not shown).

**Figure 1:**
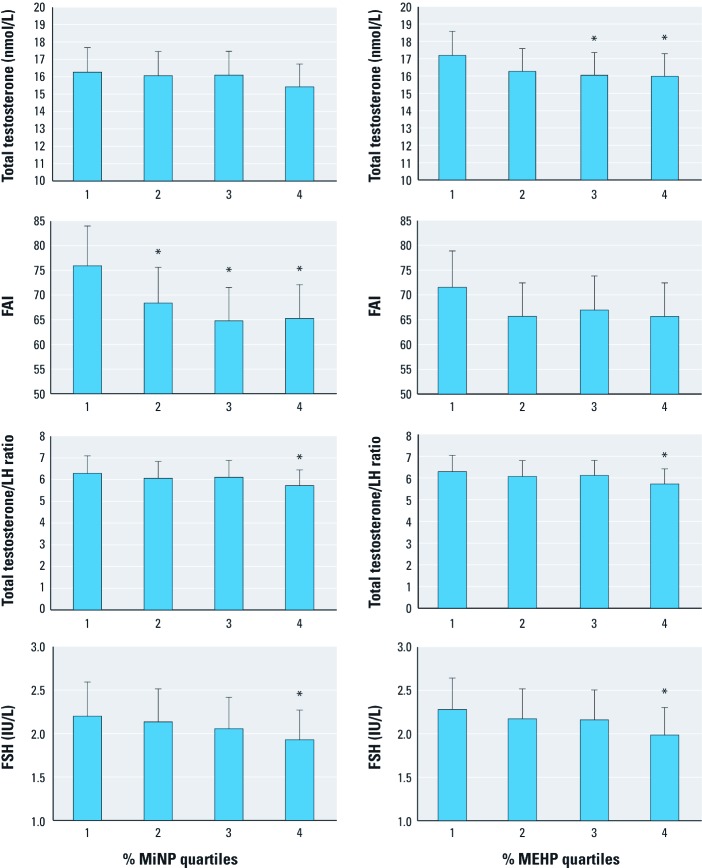
Levels of selected reproductive hormones by quartiles of %MiNP (left) and %MEHP (right); quartile 1 is lowest and 4 is highest. %MiNP quartiles (range): 1 (0.28–3.17), 2 (3.18–4.97), 3 (4.99–8.30), 4 (8.31–27.4). %MEHP quartiles (range): 1 (0.93–5.38), 2 (5.39–8.10), 3 (8.11–11.28), 4 (11.28–28.97). Scale on the *y*-axis does not start at 0; bars correspond to estimated mean values adjusted for age, BMI, smoking, alcohol intake and time of blood sample; whiskers indicate 95% CIs. *p < 0.05 compared with first (lowest) quartile of %MEHP or %MiNP.

*%MEHP and %MiNP and reproductive hormones and semen quality.* Table 4 shows associations between %MiNP and %MEHP quartiles and reproductive hormones. Figure 1 shows adjusted hormone levels by %MiNP and %MEHP quartiles. %MiNP was negatively associated with FAI, with a 15% lower (95% CI: –23, –8) FAI for the highest versus lowest quartile of %MiNP. The total testosterone/LH ratio and FAI/LH ratio were 9% lower (95% CI: –18, –0.4) and 19% lower (95% CI: –30, –8%) in the highest compared with the lowest %MiNP quartile, whereas SHBG was 10% higher in the highest quartile (95% CI: 2, 18%). Testosterone, LH, E_2_, free testosterone, free testosterone/LH and total testosterone/E_2_ were not significantly associated with %MiNP.

**Table 4:** Regression coefficients^a^ [βs (95% CIs)] for differences in ln-transformed hormones for men in the highest quartile of %MEHP (range, 11.28–28.97) or %MiNP (range, 8.31–27.38) compared with the lowest quartile of %MEHP (range, 0.93–5.38) or %MiNP (range, 0.28–3.17).

Hormone	%MEHP	p-Valueb	p-Trendc	%MiNP	p-Valueb	p-Trendc
Total testosterone (nmol/L)	–0.07 (–0.13, –0.01)	0.02	0.02	–0.05 (–0.12, 0.01)	0.09	0.11
E2 (pmol/L)	0.01 (–0.05, 0.07)	0.80	0.71	–0.02 (–0.09, 0.04)	0.49	0.78
SHBG (nmol/L)	0.01 (–0.06, 0.09)	0.74	0.96	0.10 (0.02, 0.18)	0.02	0.008
LH (IU/L)	0.01 (–0.07, 0.10)	0.75	0.51	0.04 (–0.05, 0.13)	0.40	0.45
Inhibin-B (pg/mL)	–0.02 (–0.09, 0.06)	0.66	0.77	0.03 (–0.06, 0.11)	0.57	0.54
FSH (IU/L)	–0.14 (–0.25, –0.03)	0.02	0.02	–0.13 (–0.25, –0.01)	0.04	0.03
FAI	–0.09 (–0.16, –0.01)	0.02	0.05	–0.15 (–0.23, –0.08)	< 0.001	< 0.001
Free testosterone	–0.07 (–0.12, –0.003)	0.04	0.05	–0.05 (–0.12, 0.01)	0.12	0.14
Total testosterone/LH	–0.09 (–0.17, –0.004)	0.04	0.02	–0.09 (–0.18, –0.004)	0.04	0.06
Free testosterone/LH	–0.08 (–0.17, 0.01)	0.08	0.05	–0.09 (–0.19, 0.005)	0.06	0.08
FAI/LH	–0.10 (–0.20, 0.006)	0.06	0.05	–0.19 (–0.30, –0.08)	0.001	< 0.001
Total testosterone/E2	–0.08 (–0.13, –0.03)	0.003	0.002	–0.03 (–0.08, 0.03)	0.30	0.17
Inhibin-B/FSH	0.12 (–0.04, 0.29)	0.15	0.15	0.17 (–0.02, 0.36)	0.07	0.06
aRegression coefficients adjusted for age, BMI, smoking, alcohol intake, time of day of blood sample (and assay type for inhibin-B models only). bp-Value for difference between the highest and lowest quartile. Quartiles were entered as fixed ordinate variables coded using integer values (1–4). cp-Value for linear trend across quartiles.												

%MEHP was negatively associated with total testosterone (–7% for the highest vs. lowest %MEHP quartile; 95% CI: –13, –1%), free testosterone (–7%; 95% CI: –12, –0.3%) and FAI (–9%; 95% CI: –16, –1%) (Table 4). Total testosterone/LH ratio (–9%; 95% CI: –17, –0.4%), free testosterone/LH ratio (–8%; 95% CI: –17, 1%), and FAI/LH ratio (–10%; 95% CI: –20, 0.6) were lower in the highest versus lowest quartile of %MEHP; however, the association was only significant for total testosterone/LH ratio (*p* = 0.04). SHBG, LH, and E_2_ were not associated with %MEHP, but total testosterone/E_2_ was 8% lower (95% CI: –13, –3%) in the highest compared with the lowest %MEHP quartile.

Both %MiNP and %MEHP were negatively associated with FSH, corresponding to a 13% lower (95% CI: –25, –1%) and 14% lower (95% CI: –25, –3%) in FSH in the highest versus lowest quartiles, respectively. There was no significant association with inhibin-B. Associations between inhibin-B/FSH ratio and both %MiNP and %MEHP were positive but not statistically significant; these associations were likely driven by FSH.

Trend test *p*-values were significant for all of the statistically significant highest versus lowest quartile associations in Table 4. When %MiNP and %MEHP were modeled as ln-untransformed continuous variables instead of quartiles, these associations were further confirmed (data not shown).

Table 5 shows regression analyses for semen characteristics, again entering %MiNP and %MEHP quartiles as fixed ordinate variables coded as integer values 1–4. Semen volume and the percentage of progressively motile sperm were significantly higher in the highest %MiNP quartile compared with the lowest quartile, but *p*-values for trend were not significant, and associations were not confirmed when %MiNP and %MEHP were modeled as untransformed continuous variables instead of quartiles (data not shown).

**Table 5:** Regression coefficients [βs (95% CIs)] for differences in transformed semen variables for men in the highest quartile of %MEHP (range, 11.28–28.97) or %MiNP (range, 8.31–27.38) compared with men in the lowest quartile of %MEHP or %MiNP, and *p*-values for linear trend across quartiles.

Variable	Transformation	%MEHP	*p*-Value^a^	*p*-Trend^b^	%MiNP	*p*-Value^a^	*p*-Trend^b^
Semen volume (mL)c	ln	0.04 (–0.05, 0.13)	0.37	0.28	0.14 (0.04, 0.23)	0.01	0.38
Sperm concentration (million/mL)c	Cubic root	0.11 (–0.10, 0.33)	0.31	0.23	–0.03 (–0.27, 0.21)	0.81	0.99
Total sperm count (million)c	Cubic root	0.20 (–0.12, 0.52)	0.22	0.16	0.20 (–0.15, 0.56)	0.25	0.23
Progressively motile (%)d	Squared	289 (–40, 617)	0.09	0.35	375 (20, 730)	0.04	0.18
Morphologically normal (%)e	Square root	0.11 (–0.08, 0.30)	0.27	0.21	–0.06 (–0.27, 0.15)	0.57	0.73
Total normal count (million)c	Cubic root	0.12 (–0.07, 0.31)	0.21	0.13	0.05 (–0.16, 0.27)	0.63	0.54
ap-Value for difference between highest and lowest quartile; quartiles were entered as fixed ordinate variables coded using integer values (1–4). bp-Value for linear trend across quartiles. cAdjusted for abstinence time. dAdjusted for time to semen analysis. eUnadjusted.														

%MEHP and %MiNP were correlated with each other (Spearman’s rho = 0.58, *p* < 0.001). %MiNP was only weakly correlated with ΣDiNPm (Spearman’s rho = 0.09, *p* < 0.014), and %MEHP was weakly negatively correlated with ΣDEHPm (Spearman’s rho = –0.19, *p* < 0.001).

*Phthalate metabolite levels and reproductive hormones and semen quality.* Few significant associations were detected in the final regression models of associations between reproductive hormone levels or semen quality and urinary concentrations of each individual phthalate metabolite divided into quartiles (results not shown but available on request). LH was positively associated with MnBP (9% higher LH in the highest MnBP quartile compared with the lowest, 95% CI: 1, 18%; *p* = 0.03), and this association was confirmed when MnBP was modeled as a continuous variable (data not shown). MBzP was significantly negatively associated with total sperm count in the final univariate analysis (23% lower in the highest vs. lowest MBzP quartile; 95% CI: –45, –1% *p* = 0.04), but the association was not confirmed with MBzP modeled as a continuous variable (data not shown).

There were no consistent significant associations for ΣDnBP_(i+n)_, ΣDEHPm, or ΣDiNPm with any of the reproductive hormones or semen variables (data not shown). Calculated molar sums of “low-molecular-weight phthalates” or “high-molecular-weight phthalates” or the sum of all phthalates also were not significantly associated with any hormones or semen quality variables (results not shown).

*Ethnic differences in phthalate metabolite excretion.* Participants of African (*n* = 20) or Middle Eastern origin (*n* = 32) had a higher %MEHP (mean 10.6% and 10.2%, respectively) than the European participants (mean %MEHP 8.7%) (both *p* = 0.03 for difference from Europeans). There was no significant difference in %MiNP among the ethnic groups, but only 10 participants of African origin and 22 of Middle Eastern origin had detectable levels of MiNP. Ethnicity was not a significant predictor, so it was not included in the regression models.

## Discussion

Taken together, our results point toward a compromised testosterone production combined with a pituitary–hypothalamic inhibition of gonadotropin release in men with a high %MEHP or %MiNP. These associations were consistent whether %MEHP or %MiNP were modeled as quartiles or continuous variables and robust to adjustment for various confounders; however, the possibility of chance associations due to multiple testing or effects of uncontrolled confounding cannot be ruled out.

Some diester phthalates are weakly estrogenic in *in vitro* studies (Buteau-Lozano et al. 2008; Jobling et al. 1995), but *in vivo*, phthalates have mostly shown antiandrogenic effects through inhibition of testosterone synthesis (Gray et al. 2000). If the associations observed in this study were purely the result of Leydig cell dysfunction, we would expect a concomitant rise in LH to compensate for a decrease in total testosterone, which was not the case. Rather, we observed that high %MEHP and %MiNP were associated with decreased FSH levels, which was not explained by increased estradiol or inhibin-B—two important negative feedback regulators of FSH. If phthalates do exert some estrogenic effect on the estrogen receptor, this would help explain why the association between high %MEHP or %MiNP and lower total testosterone or FAI was not accompanied by an increase in LH. Furthermore, it would offer some explanation of the observed decrease in FSH with increasing %MEHP and %MiNP. This potential dual effect of phthalates on male reproductive hormones has also been discussed by Pan et al. (2011). We further observed a positive association between SHBG and %MiNP that may be related to reduced androgen activity in men with higher SHBG as shown by Belgorosky and Rivarola (1985), or partially influenced by a possible estrogenic effect of phthalates, because both natural and synthetic estrogens have been shown to increase SHBG production in human liver cells (Hammond et al. 2008).

The observed strong correlation between %MEHP and %MiNP has not previously been described, but was expected because DEHP and DiNP share metabolic pathways. %MINP and %MEHP correlate rather weakly to ΣDEHPm and ΣDiNPm, consistent with the hypothesis that %MINP and %MEHP are individual markers of metabolism and largely independent of the level of exposure.

The primary metabolites of DEHP and DiNP (MEHP and MiNP, respectively) are thought to be the main toxicants with regard to endocrine-disrupting capacity (Chauvigné et al. 2009). Urinary concentrations of metabolites represent very recent exposure. There is considerable day-to-day variation in metabolite excretion, necessitating the use of a large number of participants in order to detect statistically significant associations because modest associations can easily be obscured by the “noise” of intraindividual variation in smaller studies. However, a single urine sample has previously been shown to be moderately predictive of each subject’s exposure over 3 months in men of reproductive age (Hauser et al. 2004). %MEHP has been proposed as a general marker of detoxification capability, with a high “setpoint” of %MEHP suggesting a poorer or slower metabolism of phthalates that might result in higher internal exposure to the active primary metabolites for a given exposure to a phthalate diester, and possibly higher internal exposure to other reproductive toxicants that share the same metabolic pathway (Hauser 2008). Intraindividual stability in phthalate metabolism, in contrast with intraindividual variability in phthalate exposures, may help explain why we found significant associations with %MEHP and %MiNP, but not with metabolite concentrations.

The participants in this study were healthy men from the general population, and their reproductive hormone levels were generally within the normal range used in our laboratory for 19-year-old men (Department of Growth and Reproduction, Copenhagen University Hospital 2012). The 15% and 9% reductions in FAI for the highest compared with the lowest quartiles of %MiNP and %MEHP may therefore seem negligible from a clinical perspective. Nevertheless, these findings suggest that a segment of the healthy male population may be vulnerable to the effects of environmental chemicals on reproductive hormone regulation.

To our knowledge, only one other study has explored associations between reproductive hormones and %MEHP. Meeker et al. (2009) found a negative association with E_2_ and a positive association with total testosterone/E_2_ ratio (*n* = 225 with calculable %MEHP), in contrast with our observations. The median %MEHP observed by Meeker et al. (2009) (10.3%) was comparable to what we observed (8.1%), but their 95th percentile for %MEHP was higher (31% vs. 17% in our study population). We observed no convincing associations of %MEHP or %MiNP with any of the semen quality variables that we studied, consistent with Hauser et al. (2006) and Herr et al. (2009). We estimated statistically significant increases in semen volume and sperm motility among men in the highest versus lowest quartile of %MiNP, but we did not observe significant linear trends across quartiles; we therefore interpret these differences as chance findings. Hauser et al. (2007) reported associations between %MEHP and sperm DNA damage, which we did not assess.

Many previous studies have measured secondary metabolites of DiNP, which are excreted in much higher concentrations than its primary metabolite MiNP (Centers for Disease Control and Prevention 2012; Wittassek 2007). The present study is, to our knowledge, the first to explore %MiNP as a marker for susceptibility to effects of phthalate exposure. MiNP was measurable in 80% of our population, which is higher than reported previously (Boas et al. 2010), due in part to the high sensitivity of the method used.

In contrast to the results for %MEHP and %MiNP, we observed only a few consistent associations between urinary levels of individual phthalate metabolites and reproductive hormone levels. Urinary MnBP concentration was positively associated with LH levels, which may have been a chance finding. This is in contrast to a number of previous studies. Meeker et al. (2009) found that urinary MEHP concentrations were negatively associated with total testosterone and E_2_, and MEHHP and MEOHP were negatively associated with FAI. The Meeker et al. (2009) population had higher urinary concentrations of MEP and DEHP metabolites, but lower MnBP and MBzP levels than our population. Men in the Meeker et al. (2009) study population, who were partners in infertile couples, also had lower total testosterone and higher LH, as well as lower inhibin-B and higher and FSH levels than men in our study population, and they had higher estradiol levels, consistent with a higher average BMI in that group. Duty et al. (2005) reported that MBzP was inversely associated with FSH in a group of infertile men. Mendiola et al. (2011) reported that FAI and free testosterone were inversely associated with DEHP metabolites and that SHBG was positively associated with MEHP in fertile men with somewhat higher urinary phthalate metabolite concentrations, but similar reproductive hormone levels compared with men in our study population. In a group of very highly exposed men, Pan et al. (2006) found an inverse association between free testosterone and urinary MnBP, and inverse associations between FSH and urinary MnBP and MEHP. In contrast, Jönsson et al. (2005) found no such associations in a smaller study of 234 men from the general population.

In the present study, we also observed no consistent patterns of associations between individual urinary phthalate metabolites and markers of semen quality, again contrasting with two previous studies of overlapping study populations (Duty et al. 2003; Hauser et al. 2006) that observed inverse associations between MnBP and sperm concentration and motility and with another smaller study where MEP was associated with decreased motility (Jönsson et al. 2005). Inconsistencies between the present study and previous studies may reflect more moderate phthalate exposures in our study population.

Use of DEHP, BBzP, and DnBP is restricted but not banned in the European Union, while use of DiNP is only restricted in certain toys and childcare articles for children < 3 years of age. Antiandrogenic effects of DiNP have been demonstrated in animal studies, albeit the effects were less potent than those reported for DBP, BBzP, and DEHP (Boberg et al. 2011). Because DiNP is considered a safe alternative to DEHP, its production and use is increasing in Europe, while DEHP production and use has declined (European Chemical Agency 2010). In our study population, DEHP exposure seemed to decrease over the study period (adjusted median ΣDEHPm levels decreased from 80 to 56 ng/mL for participants from 2007 to 2009), while DiNP exposure did not appear to change.

## Conclusions

We report that high percentages of DEHP and DiNP excreted as the primary metabolite (%MEHP and %MiNP) were associated with moderate differences in male reproductive hormones, suggesting combined effects on testicular steroidogenesis and central gonadotropin secretion. Causality can never be inferred from associations observed in this type of study, and although associations with %MEHP and %MiNP may reflect increased tissue exposures to phthalate metabolites in men with a less efficient detoxification of phthalates, %MEHP and %MiNP also may be general markers of metabolism for other chemicals that share the same metabolic pathways. Either way, our findings support a need for additional research on the possible effects of endocrine-disrupting chemicals in adults.
